# Supplementary material for: The causal role of circulating inflammatory markers in osteoporosis: a bidirectional Mendelian randomized study
Source: Front Immunol. 2024 Jul 18;15:1412298. doi: 10.3389/fimmu.2024.1412298 (PMC11291241; doi:10.3389/fimmu.2024.1412298)
Supplement: Supplementary file 1 [file DataSheet_1.zip › supplementary material/CIM-OSTEOPOROSIS /GCST90274793.csv_forest.pdf]

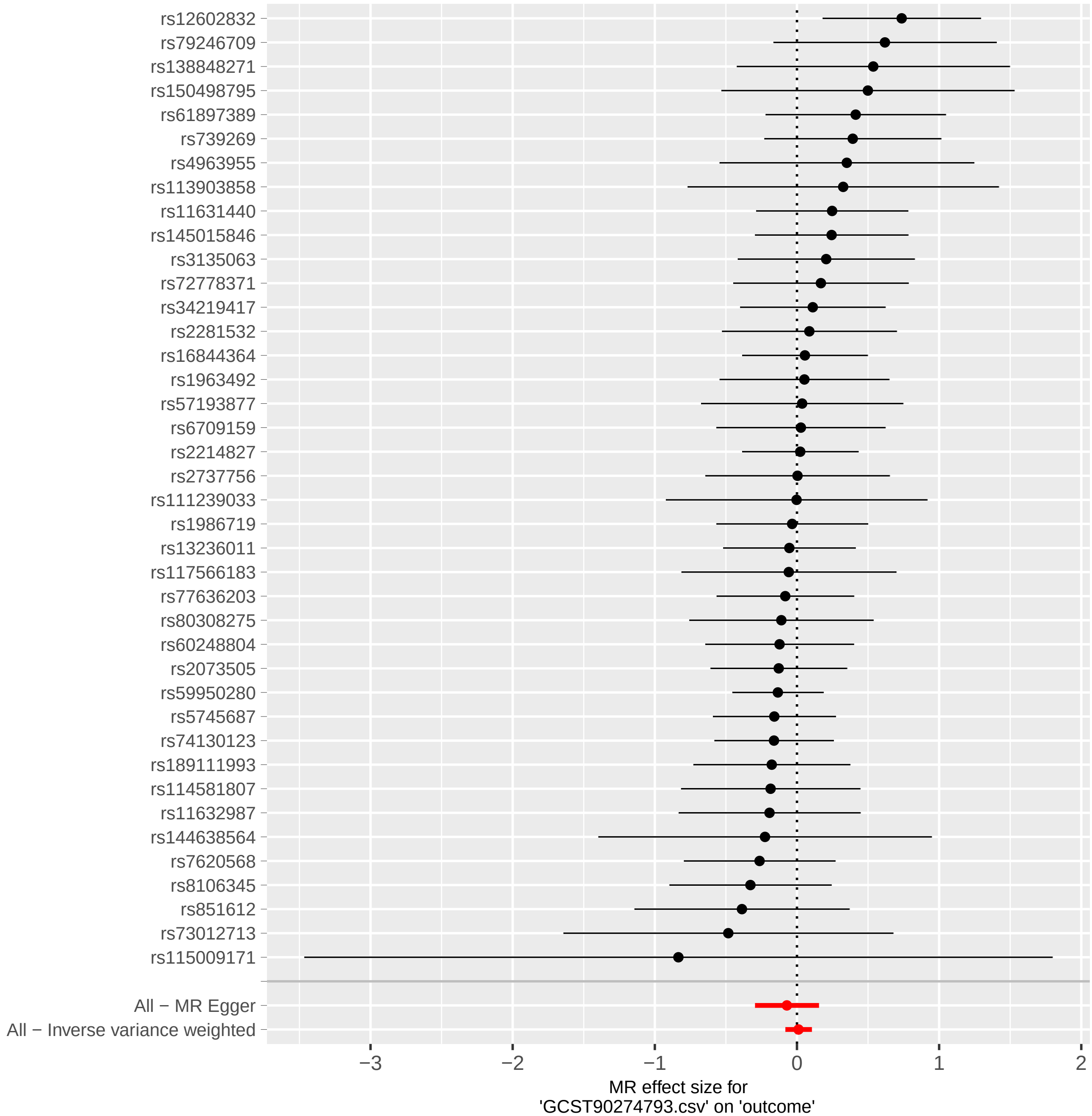
rs12602832
rs79246709
rs138848271
rs150498795
rs61897389
rs739269
rs4963955
rs113903858
rs11631440
rs145015846
rs3135063
rs72778371
rs34219417
rs2281532
rs16844364
rs1963492
rs57193877
rs6709159
rs2214827
rs2737756
rs111239033
rs1986719
rs13236011
rs117566183
rs77636203
rs80308275
rs60248804
rs2073505
rs59950280
rs5745687
rs74130123
rs189111993
rs114581807
rs11632987
rs144638564
rs7620568
rs8106345
rs851612
rs73012713
rs115009171
All − MR Egger
All − Inverse variance weighted
−3
−2
−1
0
1
2
MR effect size for
'GCST90274793.csv' on 'outcome'
